# Investigating cortical hypoxia in multiple sclerosis via time‐domain near‐infrared spectroscopy

**DOI:** 10.1002/acn3.52150

**Published:** 2024-07-22

**Authors:** Thomas Williams, Frédéric Lange, Kenneth J. Smith, Ilias Tachtsidis, Jeremy Chataway

**Affiliations:** ^1^ UCL Queen Square Institute of Neurology, University College London London UK; ^2^ Department of Medical Physics and Biomedical Engineering University College London London UK

## Abstract

**Objectives:**

Hypoperfusion and tissue hypoxia have been implicated as contributory mechanisms in the neuropathology of multiple sclerosis (MS). Our objective has been to study cortical oxygenation *in vivo* in patients with MS and age‐matched controls.

**Methods:**

A custom, multiwavelength time‐domain near‐infrared spectroscopy system was developed for assessing tissue hypoxia from the prefrontal cortex. A cross‐sectional case–control study was undertaken assessing patients with secondary progressive MS (SPMS) and age‐matched controls. Co‐registered magnetic resonance imaging was used to verify the location from which near‐infrared spectroscopy data were obtained through Monte Carlo simulations of photon propagation. Additional clinical assessments of MS disease severity were carried out by trained neurologists. Linear mixed effect models were used to compare cortical oxygenation between cases and controls, and against measures of MS severity.

**Results:**

Thirty‐three patients with secondary progressive MS (median expanded disability status scale 6 [IQR: 5–6.5]; median age 53.0 [IQR: 49–58]) and 20 age‐matched controls were recruited. Modeling of photon propagation confirmed spectroscopy data were obtained from the prefrontal cortex. Patients with SPMS had significantly lower cortical hemoglobin oxygenation compared with controls (−6.0% [95% CI: −10.0 to −1.9], *P* = 0.004). There were no significant associations between cortical oxygenation and MS severity.

**Interpretation:**

Using an advanced, multiwavelength time‐domain near‐infrared spectroscopy system, we demonstrate that patients with SPMS have lower cortical oxygenation compared with controls.

## Introduction

Multiple sclerosis (MS) is an inflammatory demyelinating disease of the central nervous system (CNS) of uncertain etiology, and a leading cause of nontraumatic neurological disability in young adults. It affects nearly 3 million people globally.^1^ Secondary progressive multiple sclerosis (SPMS) is an advanced stage of the disease characterized by the onset of progressive worsening disability following a previously relapsing–remitting course.^2^ Patients experience disability effecting mobility, balance, cognitive and visual domains.^3^ There is a paucity of effective treatment.^4^

Inflammation‐mediated neuro‐axonal injury is thought to play a key role in the neuropathology of multiple sclerosis, and there is increasing evidence that hypoperfusion and tissue hypoxia are important contributory mechanisms.^5^ Certainly, MS lesions are topographically more common in areas of hypoperfusion, and the CNS of people with MS is globally hypoperfused compared with controls.^6^, ^7^, ^8^, ^9^, ^10^, ^11^, ^12^, ^13^, ^14^, ^15^, ^16^, ^17^ The hypoperfusion is mediated, at least in part, by the release of vasoconstrictive substances such as endothelin‐1 from astrocytes and other cells.^18^, ^19^, ^20^ The importance and severity of hypoperfusion and hypoxia has been demonstrated in animal models of MS, which also show the therapeutic value of treatment with either oxygen or CNS vasodilators – which reduce both disability and the degree of demyelination.^21^, ^22^, ^23^

Near‐infrared spectroscopy (NIRS) is an optical technique which can noninvasively monitor changes in oxygenation of body tissues by relating the changes in light intensities detected by the instrument to the concentration of various chromophores, especially hemoglobin species.^24^, ^25^ The near‐infrared light can penetrate deeply into the tissue due to the relatively low absorption of the various chromophores in that optical window. Moreover, as near‐infrared light absorption is dominated by water and the oxygenated and deoxygenated forms of hemoglobin (HbO_2_ and HHb), changes in tissue oxygen saturation (StO_2_) can be determined.^25^, ^26^ Time‐domain near‐infrared spectroscopy (tdNIRS) is an advanced form of NIRS that additionally measures the arrival time of photons. The information contained in this extra temporal dimension enables the application of more refined physical models to the data processing, hence allowing the effect of the absorption and scattering properties of the tissues to be separated. Absolute quantification of chromophore concentrations can then be derived, and hence, tissue oxygen saturation is determined.^27^ The ability of tdNIRS to quantify intracranial chromophore concentrations has been validated through *in vivo* assessments, and its bedside convenience makes it an ideal tool for noninvasively investigating cortical oxygenation in people with MS.^28^, ^29^, ^30^, ^31^, ^32^

Evidence indicating that cortical oxygenation may be reduced in people with MS was first provided by an important study employing a commercial frequency‐domain NIRS (fdNIRS) system.^33^ Mean frontal oxygenation was found to be significantly lower in people with SPMS compared with controls.^33^ The previously employed fdNIRS instrument is, however, inherently limited by the fact that the concentrations of many important chromophores are estimated rather than directly calculated. Furthermore, limited information on photon pathlength is obtained from fdNIRS hence uncertainty remains regarding the exact tissues from which oxygenation data are actually obtained. In this study, we aim to address these limitations by employing a custom multiwavelength tdNIRS system capable of quantifying tissue concentrations of all relevant chromophores, and hence producing robust measurements of oxygenation.^26^, ^27^ Furthermore, by combining tdNIRS data with structural MR imaging, we are able to demonstrate with Monte Carlo simulations the precise tissues from which oxygen saturation data are obtained.

## Methods

### Participants

People with SPMS were recruited from the Queen Square Multiple Sclerosis Centre (QSMSC) at University College London. All participants were being assessed as part of the MS‐STAT2 randomized controlled trial (NCT03387670). Eligibility criteria included a confirmed diagnosis of SPMS and an Expanded Disability Status Scale (EDSS) of 4.0 to 6.5. No patients were receiving immunomodulatory DMT. Exclusion criteria included any other neurological and neurovascular conditions that might impact cortical oxygenation. Controls were eligible if they had no known neurological disease, and could be age‐matched to the patients with SPMS.

### Data collection

Following consent, demographic and medical history data were recorded, including date of birth, sex, ethnicity, medical history, smoking status, concomitant medications, blood pressure, peripheral oxygen saturations, and heart rate. Quantifications of MS clinical severity were undertaken by qualified neurologists. This assessment included EDSS, timed 25 foot walk (25FW), timed 9‐hole peg test (9HPT), symbol‐digit modalities test (SDMT), Californian verbal learning test (CVLT2), and brief visual memory test (BVMT‐R). For the assessment of photon propagation through the brain, a subset of participants were imaged on a 3T Philips Ingenia CX MR system. 3D sagittal T1‐weighted (3DT1) MPRAGE and 3D sagittal FLAIR images both with 1 mm^3^ isotropic voxels were included.

All tdNIRS recordings were obtained in a dimly lit, quiet clinic room. Scalp measurements were made to identify Fp1 and Fp2 positions, and tdNIRS emitter and detector probes were placed over these locations using a custom‐made probe holder to ensure identical source‐detector distances. This assembly was secured with elastic straps and shielded from light with a cover. The MAESTROS tdNIRS system was used to assess photon direct time of flight (DTOF) at 16 wavelengths in the near‐infrared, ranging from 780 to 870 nm in steps of 6 nm.^26^

Resting state tdNIRS recordings were obtained for 2 min. Following this, for participants undergoing functional recordings, a standardized verbal fluency task was undertaken. This task was chosen as it has previously been demonstrated to induce NIRS‐detectable activation of the prefrontal cortex.^34^ Participants were asked to name words beginning with the letter “S” for 1 min.

### NIRS data analysis

From each tdNIRS recording, between 1 and 3 observations were available from the left and right prefrontal cortex. The StO_2_ values were calculated by estimating the absolute concentration of [HbO_2_] and [HHb] in the tissue (StO_2_ = [HbO_2_]/[HbT], with [HbT] = [HbO_2_] + [HHb]). These absolute concentrations were extracted by using a spectrally constraint fitting method, using the solution of the diffusion equation for a homogenous semi‐infinite model in the forward model.^35^ The free parameters fitted in the spectrally constraint procedure were as follows: water content, lipid content, [HHb], [HbO_2_], a background parameter, and the 2 scattering parameters (a and b: scattering amplitude and scattering power, respectively). The background parameter represents potential contributions from minor tissue chromophores.^36^ Constraints were applied to the model fitting to ensure that the values remained within a physiological range. The fitting parameter boundaries are presented in Table 1. For the analysis of the changes in Hb concentrations related to the verbal fluency task, the raw tdNIRS signal was analysed using the gating method.^37^ Only the photons arriving at a late time, between 70% and 1% of the falling edge of the distribution of time of flight of photons, were considered in order to enhance the sensitivity of the measurement to changes in the gray matter.

**Table 1:** Absorption fitting parameters.

	a (cm^−1^)	b	[HbO_2_] (*μ*mol/L)	[HHb] (*μ*mol/L)	Water (%)	Lipids (%)	Background (cm^−1^)
Initial estimate	7	1	30	20	75	20	0
Lower bound	0	0	10	10	70	0	0
Upper bound	20	2	100	100	85	40	0.2

Free parameter spectral fitting was undertaken (MATLAB, “fmincon” function) to assess water, lipid, [HHb], [HbO_2_], a background parameter, and the 2 scattering parameters (a and b: scattering amplitude and scattering power, respectively). The above starting values and constraints were applied to ensure the fitted parameters remained within a physiological range.

### Modeling of photon propagation

To validate that tdNIRS data were being obtained from the prefrontal cortex, tdNIRS source and detector locations at Fp1 and Fp2 were registered with structural MRI data obtained from a single participant with SPMS. Monte Carlo simulations of photon propagation in the head were performed using the Mesh‐Based Monte‐Carlo software (https://mcx.space/).^38^ A sensitivity matrix was constructed and represented as a weighted average pathlength of all photons traversing the head, in order to demonstrate the tissues from which spectroscopy data were obtained.

### Statistical analysis

The resting state tdNIRS continuous variables of cortical oxygen saturation (StO_2_, %), total hemoglobin (HbT, *μ*mol/L), deoxyhemoglobin (HHb, *μ*mol/L), and oxygenated hemoglobin (HbO_2_, *μ*mol/L) were examined across patients with SPMS and controls. The multiple observations obtained from each participant during the same tdNIRS recording have the potential to induce pseudoreplication. To maximize use of the available data, rather than reporting an average for each participant, each individual observation was included in the analysis, but taking into account the nonindependence of the repeated sampling obtained from single participants.^39^ Mixed effect models were therefore used, with participant ID included as a random effect variable. This model included an unstructured residual covariance matrix, hence allowing different covariances between each pair of measurements on the same participant. The predictor of participant group was included as a fixed‐effect variable to assess for fixed differences between people with SPMS and controls. Due to the previously reported relationships between cerebral perfusion, cortical StO_2_ and age, participant age at data collection was included as an additional fixed‐effect covariate.^12^, ^40^ Where model assumptions were violated on the normality of residuals or homoskedasticity, distributions were calculated from bias corrected and accelerated bootstrap, clustered on the random effect of participant ID. *P*‐values are not calculated from models using bootstrapping, but may be inferred from the reported 95% confidence intervals (CI; whereby a 95% CI that does not include no effect implies a *P* < 0.05).

To assess the relationship between StO_2_ and MS severity variables, the above models were repeated, with StO_2_ as the dependent variable, and each of the MS severity variables as the predictor in multiple separate models. Age was again included as a covariate in all models, and years in education for models including cognitive performance.

The above analyses were then repeated, including each of the Hb variables (concentration of HbT, HbO_2,_ or HHb) as the dependent variable.

For the functional tdNIRS data, the change from baseline in the concentration of each chromophore was quantified as the area under the curve (AUC) across the 60 sec of the word naming task. This change was then adjusted by the initial resting state concentration; hence, the functional response represented the degree of change, over 60 sec of functional recording, relative to the resting baseline chromophore concentration. This adjusted AUC for each Hb variable was compared between participant groups and against the MS severity variables.

### Permissions and consent

This study was conducted in accordance with the Declaration of Helsinki.^41^ The study together with relevant documentation was reviewed by the National Health Service Health Research Authority Research Ethics Service (reference: 18/YH/0482), which granted a favorable opinion. All patients and controls gave written informed consent to participate.

## Results

The characteristics of the participants are shown in Table 2. The number of observations obtained was higher than the number of participants, as most patients had multiple simultaneous recordings (left and right frontal cortex, 1 to 3 detectors).

**Table 2:** Characteristics of participants recruited into the tdNIRS pilot study.

	SPMS	Controls
*n*	33	20
*N* with functional recordings	27	14
Age (median, IQR)	53.0 (49–58)	58.0 (50–64)
Sex (female)	85%	50%
Observations (resting)	101	56
Observations (functional)	82	44
EDSS (median, IQR)	6 (5–6.5)	–

IQR, interquartile range; EDSS, expanded disability status scale; people with SPMS, people with secondary progressive multiple sclerosis.

**Figure 1:**
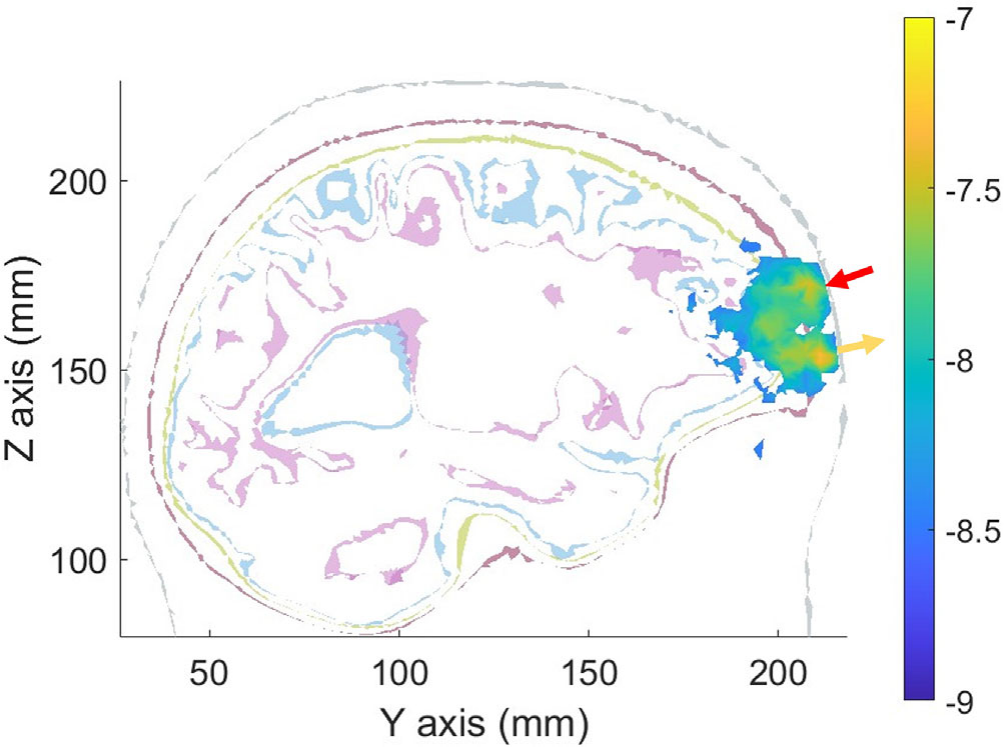
Monte Carlo simulation modeling photon propagation. tdNIRS source and detector locations at Fp1 and Fp2 were registered with structural MRI data obtained from one of the participants with SPMS. Monte Carlo simulations of photon propagation through the scalp and underlying prefrontal cortex were undertaken, and the sensitivity matrix of the measurement is represented here as a weighted average pathlength of all photons traversing the head. tdNIRS, time‐domain near‐infrared spectroscopy; SPMS, secondary progressive MS.

The results of modeling photon propagative between emitter and detector in a single subject with SPMS are shown in Figure 1. This demonstrates good penetrance of photons into the prefrontal cortex, confirming that tdNIRS data are obtained from the CNS.

### Comparison of cortical oxygenation between groups

**Figure 2:**
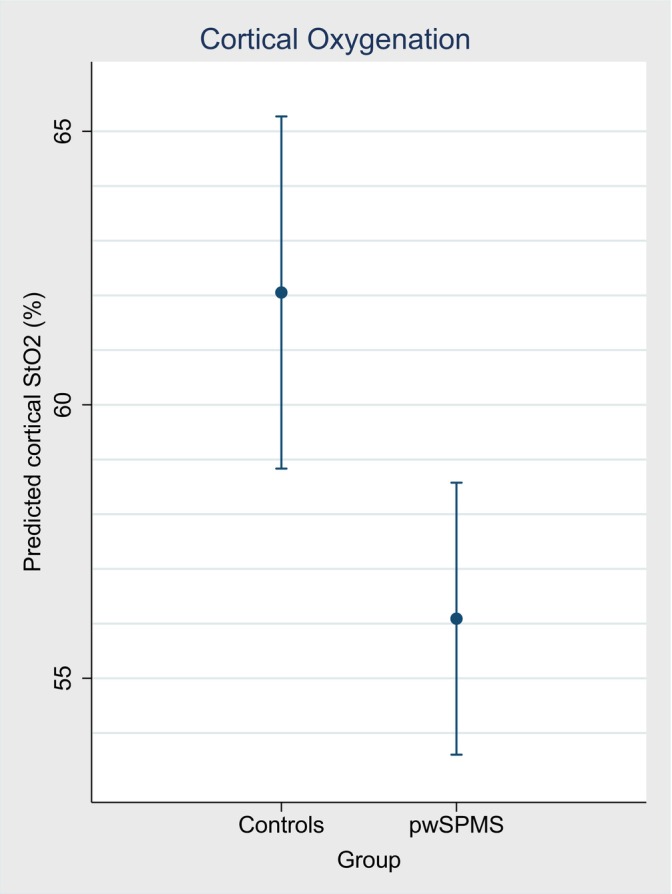
Modeled cortical oxygenation data in patients with SPMS and age‐matched controls. Predicted marginal means and 95% CI for the repeated measures cortical StO_2_ (%) between patients with SPMS and age‐matched controls. The data represent that also shown in Table 3. CI, confidence interval; StO_2_, cortical oxygen saturation.

The modeled cortical StO_2_ between people with SPMS and controls, adjusted for age, is shown in Figure 2 with the predicted margins for each group shown in Table 3. Cortical StO_2_ was significantly lower in people with SPMS compared with controls (−6.0% [95% CI: −10.0 to −1.9], *P* = 0.004).

**Table 3:** Predicted margins for modeled cortical oxygenation in each group.

	Controls	SPMS	Beta‐coefficient [95% CI], SPMS relative to controls
Cortical StO_2_ (%)	62.1 [58.8 to 65.3]	56.1 [53.6 to 58.6]	−6.0 [−10.0 to −1.9], *P* = 0.004

Predicted marginal means and 95% CI for the repeated measures cortical StO_2_ between participant groups, adjusted for age. [], 95% CI; StO_2_, cortical oxygen saturation; CI, confidence interval; SPMS, people with secondary progressive multiple sclerosis.

### Relationships between cortical oxygenation and MS severity variables

The relationships between cortical StO_2_ and MS severity variables, while adjusting for age, are shown in Table 4. There were no statistically significant relationships between MS severity variables and cortical StO_2_.

**Table 4:** The relationship between MS severity variables and cortical oxygenation.

MS severity variable	Coefficient against cortical StO_2_ (%)
MS duration (years)	+0.12 [−0.02 to +0.34]
EDSS	−1.14 [−3.43 to +0.94]
25FW speed (mean, ft/sec)	+0.57 [−1.22 to +2.00]
9HPT speed (mean, sec^−1^)	−278.04 [−643.51 to +68.69]
SDMT	−0.13 [−0.36 to +0.07]
CVLT2	−0.15 [−0.40 to +0.04]
BVMT‐R	−0.05 [−0.29 to +0.19]

Data represent the coefficients and 95% CI between StO_2_ (dependent variable) and the MS severity variables (include in separate models as the predictor). In all analyses, age was included as a covariate, and estimates were obtained through bias corrected and accelerated bootstrap, clustered on patient ID. P‐values are therefore not reported, but can be inferred from the 95% CI – whereby if the CI includes zero, the data do not support a significant relationship. For measures of cognitive performance, years in education was additionally included as a covariate. CI, confidence interval; EDSS, expanded disability status scale; 25FW, timed 25 foot walk, speed; 9HPT, timed 9‐hole peg test, speed; SDMT, symbol‐digit modalities test; CVLT2, Californian verbal learning test; BVMT‐R, brief visual memory test, revised; StO_2_, cortical oxygen saturation.

### Relationships between tissue hemoglobin concentration, participant groups, and MS severity

The modeled cortical [Hb] between participant groups and the relationship between cortical [Hb] and measures of MS severity are shown in Tables 5 and 6. HbO_2_ was lower in SPMS compared with controls (−7.1 μmol/L [95% CI: −11.4 to −2.8], *P* = 0.001). Higher EDSS demonstrated a modest relationship with higher HbT: For each point increase in EDSS, HbT increased by +5.13 μmol/L [95% CI: +0.70 to +10.06].

**Table 5:** Predicted margins for modeled cortical hemoglobin concentrations in each group.

	Controls	SPMS	Beta‐coefficient [95% CI], SPMS relative to controls
HbT (*μ*mol/L)	70.0 [64.2 to 75.8]	65.3 [61.0 to 69.7]	−4.6 [−11.9 to +2.7], *P* = 0.213
HbO_2_ (*μ*mol/L)	43.1 [39.6 to 46.6]	36.0 [33.4 to 38.6]	−7.1 [−11.4 to −2.8], *P* = 0.001
HHb (*μ*mol/L)	27.0 [23.4 to 30.7]	29.3 [26.6 to 32.0]	+2.3 [−2.3 to +7.0], *P* = 0.331

Predicted marginal means and 95% CI for the repeated measures cortical [Hb] variables between participant groups. In all cases, age is included as a covariate. [], 95% CI; CI, confidence interval.

**Table 6:** The relationship between MS severity variables and cortical hemoglobin concentration.

MS severity variable	Coefficient against HbT (*μ*mol/L)	Coefficient against HbO_2_ (*μ*mol/L)	Coefficient against HHb (*μ*mol/L)
MS duration (years)	0.08 [−0.45 to +0.62]	0.11 [−0.17 to +0.38]	−0.04 [−0.38 to +0.29]
EDSS	5.13 [+0.70 to +10.06]	1.96 [−0.75 to +4.87]	2.94 [−0.06 to +5.24]
25FW speed (ft/sec)	−0.45 [−5.03 to +4.14]	0.09 [−2.30 to +2.49]	−0.37 [−3.26 to +2.52]
9HPT speed (sec^−1^)	243.41 [−523.92 to +1010.73]	−108.84 [−507.99 to +290.32]	341.81 [−131.36 to +814.98]
SDMT	0.11 [−0.39 to +0.60]	−0.05 [−0.31 to +0.20]	0.15 [−0.16 to +0.46]
CVLT2	0.05 [−0.36 to +0.47]	−0.08 [−0.29 to +0.13]	0.13 [−0.13 to +0.40]
BVMT‐R	0.01 [−0.72 to +0.73]	−0.06 [−0.43 to +0.31]	0.04 [−0.43 to +0.53]

Data represent the coefficients and 95% CI between cortical [Hb] variables (dependent variable) and the MS severity variables (include in separate models as the predictor). In all analyses, age and hematocrit were included as covariate. For measures of cognitive performance, years in education was additionally included as a covariate. For models including HbT as the dependent variable, estimates were obtained through bias corrected and accelerated bootstrap, clustered on patient ID. *P*‐values are therefore not reported, but can be inferred from the 95% CI – whereby if the CI includes zero, the data do not support a significant relationship. EDSS, expanded disability status scale; 25FW, timed 25 foot walk, speed; 9HPT, timed 9‐hole peg test, speed; SDMT, symbol‐digit modalities test; CVLT2, Californian verbal learning test; BVMT‐R, brief visual memory test, revised; HbT, total hemoglobin concentration; HbO_2_, oxygenated hemoglobin concentration; HHb, deoxygenated hemoglobin concentration. [], 95% CI; CI, confidence interval.

### Relationships between change in hemoglobin concentration during cognitive stimulation, participant groups, and MS severity

The changes in hemoglobin concentrations (ΔHbT, ΔHbO2,and ΔHHb) during the cognitive stimulation task are shown in Table 7, expressed as change in μmol/L per unit of resting hemoglobin concentration. Table S1 reports the relationships between the same hemoglobin data and measures of MS severity. There was insufficient evidence to support a difference in cortical activation patterns between people with SPMS and age‐matched controls. There was also no clear evidence for any relationships between the ΔHb variables and measures of MS severity.

**Table 7:** Predicted margins for modeled change in cortical hemoglobin concentration in cases and controls.

	Controls (*n* = 14)	SPMS (*n* = 27)
ΔHbT (*μ*mol/L)	0.64 [0.24 to 1.05]	0.49 [0.31 to 0.66]
ΔHbO2 (*μ*mol/L)	0.90 [0.10 to 1.69]	1.06 [0.52 to 1.60]
ΔHHb (*μ*mol/L)	0.32 [−0.18 to +0.81]	−0.16 [−0.54 to +0.23]

Predicted marginal means and 95% CI for the repeated measures cortical ΔHb variables between participant groups. ΔHb variables were calculated as the area under the curve for the first 60 sec of the cognitive simulation task, divided by the resting baseline [Hb]. They therefore represent the change in [Hb], over 60 sec, relative to the baseline [Hb]. In all analyses, age was included as a covariate. HbT, total hemoglobin concentration; HbO_2_, oxygenated hemoglobin concentration; HHb, deoxygenated hemoglobin concentration. [], 95% CI; CI, confidence interval.

## Discussion

We have investigated the feasibility of collecting tdNIRS data from a cohort of people with SPMS. We have demonstrated the tissues from which our equipment collects data by modeling photon paths through structural MR imaging, and then explored the differences in tdNIRS data between cases and controls, and against established measures of MS severity. Our main finding is that people with SPMS appear to have lower cortical oxygen saturations than age‐matched controls.

Our findings support those previously reported by Yang and Dunn, although with some differences in detail.^33^ Our modeled cortical StO_2_ of 56.1% [95% CI: 53.6–58.6] is a little higher than that reported by Yang and Dunn in people with SPMS (52.6, SD: 9), perhaps because their mean results included three participants with very low cortical StO_2_. The Yang and Dunn recordings were also made in Calgary, at an altitude of 1000 m, while our recordings were taken near sea level in London. It is unclear whether this difference in altitude is sufficient to contribute to any small differences in values between the two studies. The concentration of HbO_2_, HHb, and HbT that we report is higher than those reported by Yang and Dunn – which likely relates to differences in the NIRS systems used. The fdNIRS technique used by Yang and Dunn cannot quantify the concentrations of other chromophores which contribute to light attenuation. For example, the contribution of lipid was not quantified by Yang and Dunn, and water was assumed to be 75% of tissue in all participants. In our participants, water was calculated to range from 71.6% to 77.5% of tissue and lipid from 16.6% to 22.8%. It should be noted, however, that despite the differences in technique used, the control cortical oxygenation values (62.1% compared to 63.5%) are very similar in both studies – providing some validation between frequency‐domain and time‐domain NIRS techniques.

Despite demonstrating that people with SPMS had lower cortical oxygenation in this cohort compared to age‐matched controls, and in contrast to the previous fdNIRS report by Yang and Dunn, we did not find any clear evidence to support a relationship between tdNIRS variables and measures of MS severity.^33^ Firstly, it should be acknowledged that MS is a spatially heterogenous disease, and as such, our measurements taken from the prefrontal cortex may not be representative of disease severity elsewhere in the CNS. Outcome measures quantifying walking ability (EDSS, 25FW) are particularly influenced by spinal cord demyelination, and cognitive measures such as the SDMT are associated with the overall brain lesion load.^42^ As such, the absence of a relationship between prefrontal cortical oxygenation and these disability measures is not unexpected. As a relatively small study, we also have limited statistical power to demonstrate any such significant relationships.

Additionally, the relationships between measured cortical hemoglobin oxygenation and the neuropathological processes occurring in MS are likely to be complex. The cortical oxygenation signal obtained from tdNIRS includes contributions from all contained blood vessels, each of which will be expected to contain hemoglobin of differing oxygen saturations. Changes in the volume of the arterial, venous, or capillary compartments will therefore result in changes in the acquired cortical oxygenation signal, as will changes in tissue oxygen utilization.^43^ Preclinical and clinical data have suggested that restricted perfusion may be a potential source of pathogenic hypoxia in MS.^6^, ^7^, ^8^, ^10^, ^11^, ^12^, ^13^, ^14^, ^15^, ^16^, ^17^, ^21^, ^22^, ^23^ While restricted perfusion may lead to reduced cortical oxygenation, as reported in the examples of intra‐operative carotid endarterectomy or post‐SAH vasospasm, it may also initially be countered by autoregulation‐mediated arterial vasodilation.^29^, ^30^, ^44^ By increasing the volume of the arteriolar compartment, StO_2_ may therefore actually be increased until perfusion is restricted to the extent that cerebral blood flow declines. Similarly, if the neuropathology of MS results in a reduced cortical metabolic rate, as has been implicated previously, reductions in tissue oxygen utilization may also contribute to the observed cortical StO_2_.^45^ The potential complexity of factors contributing to cortical StO_2_ signal is highlighted by the finding of increased cortical oxygenation over the hemisphere of an established cerebral infarct, compared with the contralateral hemisphere, emphasizing the important role of oxygen utilization in StO_2_.^28^

Overall, our main finding of lower cortical oxygenation in people with SPMS compared with controls is therefore supportive of a potential role for hypoxia and hypoperfusion in the pathogenesis of SPMS. There is, however, likely to be a complex interplay of multiple physiological and pathophysiological processes contributing to the cortical oxygenation signal, which will require larger, longitudinal studies to delineate further.

### Relationship between functional NIRS variables and MS severity

Our comparison of functional NIRS data between cases and controls did not find significant differences, and no relationship was found with MS severity variables. Again, the relationship between functional changes in cortical NIRS parameters, reflecting the degree of cortical activation, may involve a complex interaction of multiple physiological responses. While there are few published studies of functional NIRS data in MS, two reports have suggested that increasing disability may be associated with greater cortical activation, either during a walking task or during cognitive stimulation.^46^, ^47^ These results have been supported by observations in people with Parkinson's disease, whereby increased cortical activation was noted compared with controls during a walking task.^48^ Similar results have also been found using EEG techniques, with increased cortical activation in people with MS when walking.^49^ Rather than being limited by pathologically impaired perfusion, and hence declining in SPMS, we therefore speculate that the amplitude of a functional NIRS responses may instead be more closely related to the degree of cognitive effort required, hence increasing when physical or cognitive disability make a task more difficult for the patient to complete. Further research using functional NIRS recordings across a broader range of MS severity would be required to investigate this.

An advantage of our multiwavelength tdNIRS system is that it can be optimized to explore additional chromophore concentrations, beyond hemoglobin. Given the identified complexities in the interpretation of cortical StO_2_, future investigations into the concentration of the oxidative states of cytochrome C‐oxidase (CCO) may permit greater insight into the pathophysiology of SPMS. As complex IV of the mitochondrial electron transport chain, the oxidative state of CCO provides a measure of mitochondrial ATP production.^50^, ^51^ Measured alongside StO_2_, CCO would therefore allow a more direct measure of cortical activation and metabolism. Changes in the cortical oxidative state of CCO in response to therapies aimed at improving CNS perfusions, as has already been demonstrated in animal models of MS, would additionally be an attractive outcome measure for early interventional trials.^23^

### Limitations and future work

Limitations include those related to the study design and those related to the tdNIRS technique. Firstly, the study design was limited by the relatively small number of participants and absence of longitudinal data collection. The data reported here should therefore be viewed as exploratory and used to refine hypotheses for testing in larger longitudinal datasets. Limitations of the tdNIRS technique principally relate to the lack of spatial resolution and the limited depth from which signal can be obtained. We have clarified this through our demonstration of the tissues from which our data were derived through statistical modeling of photon paths, and similar approaches, combined with structural MRI data, could be used to apply tdNIRS to other recording sites. Future work should therefore look to explore longitudinal relationships between tdNIRS parameters and measures of disease severity in larger cohorts of people with MS and controls, and to refine the model used to process the tdNIRS data. Furthermore, the potential to incorporate measures of mitochondrial function (CCO), and the utilization of MRI data to confirm the topography of the tdNIRS recordings, provides an exciting opportunity for further refinement of our techniques.

## Conclusions

We have demonstrated that people with SPMS have lower resting cortical StO_2_ compared with age‐matched controls using an advanced, multiwavelength tdNIRS system. This system provides further support for the potential role of hypoxia in the pathophysiology of progressive MS and encourages further research into the application of NIRS techniques in people with MS.

## Author contributions

TW: involved in study conception, design, obtaining ethical approval, patient recruitment, patient assessments, statistical analysis, and drafting and revisions of manuscript. FL involved in study conception and design, development and maintenance of spectroscopy equipment, patient assessments, spectroscopy data analysis, photon pathlength analysis, and revisions of manuscript. KS involved in study conception and design, and revisions of manuscript. IT involved in study conception and design, development and maintenance of spectroscopy equipment, and revisions of manuscript. JC served as a chief investigator of MS‐STAT2 trial, and involved in acquiring funding, study conception and design, and revisions of manuscript.

## Conflict of interest

TW: has received honorarium for educational talks from Novartis and Merck; his fellowship was funded by the MS‐STAT2 clinical trial grant. FL: No relevant conflicts. IT: No relevant conflicts. KS: No relevant conflicts. JC: In the last 3 years, JC has received support from the Health Technology Assessment (HTA) Programme (National Institute for Health Research, NIHR), the UK MS Society, the US National MS Society and the Rosetrees Trust. He is supported in part by the NIHR University College London Hospitals (UCLH) Biomedical Research Centre, London, UK. He has been a local principal investigator for a trial in MS funded by the Canadian MS society. A local principal investigator for commercial trials funded by Ionis, Novartis and Roche; and has taken part in advisory boards/consultancy for Azadyne, Biogen, Lucid, Janssen, Merck, NervGen, Novartis, and Roche.

## Supporting information


**Table S1.** The relationship between MS severity variables and change in cortical haemoglobin concentration during stimulation task.

## Data Availability

The data that support the findings of this study are available from the corresponding author upon reasonable request.
