# Supplementary material for: Investigating cortical hypoxia in multiple sclerosis via time‐domain near‐infrared spectroscopy
Source: Ann Clin Transl Neurol. 2024 Jul 22;11(9):2372–81. doi: 10.1002/acn3.52150 (PMC11537135; doi:10.1002/acn3.52150)
Supplement: Supplementary file 1 — Table S1. The relationship between MS severity variables and change in cortical haemoglobin concentration during stimulation task. [file ACN3-11-2372-s001.docx]

Table S1: The relationship between MS severity variables and change in cortical haemoglobin concentration during stimulation task

| MS severity variable | $\Delta HbT$ ($\mu$M) | $\Delta HbO2$ ($\mu$M) | $\Delta HHb$ ($\mu$M) |
| --- | --- | --- | --- |
| Word naming performance (words named) | -0.01  -0.03 to +0.03 | -0.00  -0.09 to +0.09 | -0.02  -0.08 to +0.04 |
| MS duration (y) | -0.01  -0.02 to +0 .00 | -0.01  -0.04 to +0.03 | -0.01  -0.04 to +0.02 |
| EDSS | +0.03  -0.12 to +0.15 | +0.01  -0.45 to +0.47 | +0.11  -0.31 to +0.53 |
| 25FW speed (ft/s) | -0.04  -0.18 to +0.09 | -0.08  -0.42 to +0.25 | -0.02  -0.33 to +0.29 |
| 9HPT speed (s^-1^) | -20.73  -40.33 to -0.57 | -40.70  -96.17 to +14.77 | -7.66  -60.59 to +45.28 |
| SDMT | -0.01  -0.02 to +0.00 | -0.01  -0.05 to +0.02 | -0.01  -0.04 to +0.03 |
| CVLT2 | +0.00  -0.01 to +0.01 | +0.01  -0.02 to +0.04 | -0.01  -0.04 to +0.02 |
| BVMT-R | +0.00  -0.01 to +0.01 | +0.00  -0.05 to +0.04 | -0.00  -0.05 to +0.04 |

Data represent the coefficients and 95% confidence interval between cortical $\Delta Hb$ variables (dependent variable), and the MS severity variables (include in separate models as the predictor). In all analyses, age was included as a covariate. For measures of cognitive performance, years in education was additionally included as a covariate. EDSS, expanded disability status scale; 25FW, timed 25 foot walk, speed; 9HPT, timed 9-hole peg test, speed; SDMT, symbol-digit modalities test; CVLT2, Californian verbal learning test; BVMT-R, brief visual memory test, revised; HbT, total haemoglobin concentration; HbO_2_, oxygenated haemoglobin concentration; HHb, deoxygenated haemoglobin concentration. [ ] = 95% confidence interval
